# Awareness of Menopause and Hormone Replacement Therapy Among Women in a Tertiary Care Hospital in Tamil Nadu

**DOI:** 10.7759/cureus.98819

**Published:** 2025-12-09

**Authors:** Aswini G, Veena SR, Meena T S, Geetha Lakshmi

**Affiliations:** 1 Obstetrics and Gynaecology, Sree Balaji Medical College and Hospital, Chennai, IND

**Keywords:** health - knowledge, hormone-replacement therapy, knowledge attitudes and practices (kap), post menopause, women’s health

## Abstract

Background

Menopause represents a crucial physiological milestone in a woman’s life, often associated with multiple physical and emotional changes. However, awareness regarding menopause and hormone replacement therapy (HRT) remains inadequate in many parts of India. This study aimed to assess the awareness, knowledge, and attitudes toward menopause and HRT among women attending a tertiary-care hospital in Tamil Nadu.

Methods

A hospital-based cross-sectional study was conducted among 76 women aged 40 years and above at the Department of Obstetrics and Gynaecology, Sree Balaji Medical College and Hospital, Chennai. Data were collected using a pretested structured questionnaire that included socio-demographic details, menstrual history, awareness of menopause, and perceptions regarding HRT. Descriptive statistics were applied to summarize the findings, and chi-square tests were used to assess associations between awareness and socio-demographic characteristics. Logistic regression identified independent predictors of HRT awareness. A p-value <0.05 was considered statistically significant.

Results

The majority of participants were aged 46-50 years (30, 39.5%), followed by >50 years (26, 34.2%) and 40-45 years (20, 26.3%). Among the respondents, 63 (82.9%) had heard of menopause, and 54 (71.1%) were aware of its symptoms. However, only 27 (35.5%) women were aware of HRT. Awareness was significantly higher among graduates and above (15, 48.4%) compared to illiterate women (one, 16.7%) (p = 0.013). Similarly, employed women (17, 60.7%) demonstrated greater awareness than unemployed women (10, 20.8%) (p = 0.011). Multivariate logistic regression showed that higher education (adjusted odds ratio (AOR) 0.24, 95% CI 0.08-0.76, p = 0.015) and employment (AOR 0.28, 95% CI 0.10-0.79, p = 0.018) were independent predictors of HRT awareness. A majority (49, 64.5%) viewed menopause as a phase of maturity, while 30 (39.5%) believed it marked the end of sexual life. Nearly half (36, 47%) were uncertain about HRT safety.

Conclusion

Although awareness of menopause was relatively high (82.9%), understanding and acceptance of HRT were low (35.5%). Education and employment significantly influenced awareness levels. Strengthening health education, counseling, and community-based programs is essential to improve menopausal literacy and empower women to make informed health decisions.

## Introduction

Menopause is defined as the permanent cessation of menstruation when ovarian follicular activity ends, typically confirmed after 12 consecutive months of amenorrhea. This transition from reproductive to nonreproductive life is accompanied by profound hormonal shifts, especially in estrogen and progesterone, that influence multiple systems in the body. Clinically, menopause is not simply a gynecologic event but a systemic change affecting bone, cardiovascular, metabolic, and neurocognitive health. The health implications of estrogen deficiency in the postmenopausal state include increased risk of osteoporosis, unfavorable lipid profiles, and greater incidence of cardiovascular disease, among others [1]. Biologically, menopause is conceptualized as a neuroendocrine adaptation to falling ovarian reserve, with feedback alterations in gonadotropins and changing sensitivity of peripheral tissues to sex steroids [2].

Globally, the demographic trend toward aging populations means that an increasing number of women spend a significant portion of their lives beyond menopause. The rising life expectancy, combined with lower fertility rates, ensures that menopause, and its sequelae, are becoming a major public health priority. Yet despite this, awareness of menopausal health and its management remains suboptimal in many settings. In Jordan, for example, less than half of premenopausal women had adequate knowledge of menopausal symptoms or available treatments, and awareness of hormone therapy was particularly low [3]. In a fourth-tier city in China, awareness levels of menopausal hormone therapy were likewise poor, highlighting that even in urbanized Asia, gaps in knowledge persist [4]. Research from the United Arab Emirates demonstrated that a majority of women lacked correct understanding of menopause and its therapeutic options, with pervasive misconceptions [5].

The discourse around hormone therapy (HT) itself adds complexity. Surveys in European settings suggest that despite clinical evidence, women and doctors often hesitate to discuss or use HT due to lingering concerns about safety and conflicting guidelines [6]. In the United States, determinants of knowledge and current use of hormone replacement therapy (HRT) are strongly associated with socioeconomic factors, access to care, and health literacy [7]. Attitudinal studies have shown that women’s beliefs about menopause and hormone therapy are heavily shaped by cultural narratives, social norms, and available information channels [8]. In Spain, the persistent dilemma of whether to initiate therapy reflects enduring knowledge gaps and risk-benefit uncertainty on both sides - patients and providers [9]. Physician awareness of patient attitudes has also been studied, and findings indicate that clinicians may misjudge how much their patients know or fear about therapy [10]. More recently, in Kuala Lumpur, a community survey found that although many women had heard of menopause, fewer than 40% had accurate knowledge of hormone therapy’s advantages and risks, and informal media sources often dominated their understanding [11]. In Saudi Arabia’s Taif City, over 70% of women had no formal knowledge of hormone therapy, and only a small fraction recognized its role in preventing osteoporosis [12].

In India, awareness and uptake of menopause-related care and hormone therapy remain underexplored. Recent Indian data reflect a limited but growing attention to the topic. In a multi-stakeholder study spanning consumers and healthcare providers, awareness of menopause and hormone therapy was found to be modest and patchy across regions and clinical settings [13]. Another investigation of obstetricians and gynecologists highlighted gaps in knowledge, varying attitudes, and inconsistent practices regarding menopause management in India [14]. Despite increasing research interest, there remains a dearth of community‐based or hospital‐based studies in many Indian states quantifying women’s awareness, perceptions, and barriers related to hormone therapy.

Within Tamil Nadu, although healthcare infrastructure and literacy are relatively robust compared to many Indian states, there is limited published data on menopausal awareness and hormone therapy acceptance among women attending tertiary centers. Understanding the level of knowledge, prevailing misconceptions, and readiness for therapy is essential, especially in tertiary care settings where women present for a wide array of health services. Women in such settings may have better access to health information, yet may also harbor silent gaps in understanding or be influenced by myths that deter effective care.

By focusing on women attending a tertiary care hospital in Tamil Nadu, this study aims to elucidate the current awareness, knowledge, and perceptions regarding menopause and hormone therapy. It will help identify which symptoms are commonly known or unknown, which risks or benefits of hormone therapy are misunderstood, and what barriers women perceive toward therapy adoption. The findings are expected to guide culturally sensitive educational interventions, strengthen physician-patient communication in menopausal care, and inform policy efforts to incorporate menopausal health into broader women’s health frameworks.

## Materials and methods

Methodology

Study Design and Setting

The present investigation was designed as a hospital-based cross-sectional study carried out in the Department of Obstetrics and Gynaecology, Sree Balaji Medical College and Hospital (SBMCH), Chennai, Tamil Nadu. The study was conducted over a period of three months, from June 2025 to August 2025. Sree Balaji Medical College is a tertiary-care teaching hospital serving a large catchment area, including both urban and semi-urban populations. This setting was ideal for assessing women’s awareness, perceptions, and practices regarding menopause and HRT, as it provided access to women of diverse educational and socioeconomic backgrounds. Prior to initiation, ethical clearance was obtained from the Institutional Human Ethics Committee of SBMCH (002/SBMCH/IHEC/2025/2534). 

Study Population

The study population comprised women aged 40 years and above who attended the outpatient and inpatient departments of Obstetrics and Gynaecology during the study period. Both perimenopausal and postmenopausal women were eligible for inclusion. These women represented the age group most likely to experience menopausal transition and related health changes, making them appropriate subjects for assessing awareness and attitudes toward menopause and its management. Participants were recruited consecutively from patients visiting for general gynaecological complaints, routine health check-ups, or menopausal symptoms. Women who met the eligibility criteria and provided informed consent were enrolled. Exclusion criteria included women who were unwilling to participate, those with cognitive impairment that could hinder comprehension or recall, and individuals with serious comorbid conditions such as psychiatric illness or neurological disorders that could compromise reliable participation. This careful selection ensured that participants could respond meaningfully to the questionnaire items.

Inclusion and Exclusion Criteria

Women aged 40 years and above who were in the perimenopausal or postmenopausal phase, as determined by their menstrual history and clinical symptoms, were included in the study. They were recruited from both outpatient and inpatient services of the Obstetrics and Gynaecology department. Only those who gave written informed consent were considered eligible. Women with prior abdominal or pelvic surgeries other than caesarean section, those diagnosed with hormone-dependent malignancies, or those with endocrine or psychiatric disorders were excluded. Patients who were unable to comprehend the study questions or unwilling to continue participation were also excluded. These criteria ensured a uniform study population and minimized confounding variables that could affect awareness or perception levels.

Sample Size Determination

The sample size was calculated using the standard formula for cross-sectional studies: n = Z² × P(1-P) / d². Assuming a 95% confidence level (Z = 1.96), a presumed prevalence (P) of 60% awareness regarding menopause and HRT, and an allowable error (d) of 11%, the estimated minimum sample size was calculated as 76 participants. A convenient sampling technique was adopted to recruit eligible women attending the hospital during the data collection period. This pragmatic approach was chosen due to the limited duration of the study and the availability of participants within the hospital setting.

Data Collection Instrument

Data collection was performed using a pretested, structured questionnaire developed after a comprehensive literature review and consultation with subject experts in obstetrics, gynaecology, and public health. The questionnaire was prepared in English and translated into Tamil for better comprehension among participants. It underwent pre-validation by three domain experts to ensure content accuracy, clarity, and cultural appropriateness. A pilot study was conducted on ten women outside the study cohort to assess feasibility and internal consistency, following which necessary modifications were incorporated. The questionnaire demonstrated strong content validity, confirmed through expert review and alignment with established literature. Face validity was ensured by pretesting with participants for clarity and comprehension. The pilot study yielded a Cronbach’s alpha of >0.70, indicating acceptable internal consistency and reliability. Construct validity was supported by consistent responses across related domains during pilot evaluation.

The final questionnaire (Appendix) consisted of five sections encompassing socio-demographic data, menstrual and reproductive history, knowledge and awareness about menopause, attitudes toward menopausal changes, and practices related to hormone replacement therapy. The items covered understanding of menopausal symptoms, perceived physical and psychological changes, awareness of health risks such as osteoporosis and cardiovascular disease, and perceptions regarding the safety and role of HRT. In this study, awareness refers to a woman’s knowledge, understanding, and recognition of menopause and HRT. This includes her ability to identify menopausal symptoms, understand the physiological changes associated with menopause, and demonstrate familiarity with the purpose, benefits, risks, and indications of HRT. The majority of questions were close-ended with multiple-choice options, designed to ensure uniformity of responses and ease of statistical analysis.

Data Collection Procedure

Eligible participants were approached during their routine outpatient or inpatient visits after a brief screening for eligibility. The investigator explained the purpose and objectives of the study in detail, either in English or Tamil, depending on the participant’s preference. Written informed consent was obtained prior to inclusion. Each participant was interviewed individually in a private setting to ensure comfort and confidentiality. The interviews were conducted face-to-face by the principal investigator using the structured questionnaire. The average duration of each interview was approximately 15 to 20 minutes.

The investigator ensured that participants clearly understood each question, and explanations were provided in simple terms wherever necessary. The interviews were non-judgmental and supportive in tone to encourage open responses. No invasive or diagnostic procedures were performed as part of the study, and there were no physical or financial risks involved. All collected data were anonymized immediately by assigning unique identification codes, and no personal identifiers were retained. The completed questionnaires were securely stored in locked files accessible only to the research team, ensuring complete confidentiality.

Ethical considerations

The study protocol was approved by the Institutional Human Ethics Committee (002/SBMCH/IHEC/2025/2534). Participation was entirely voluntary, and informed consent was obtained from all participants before data collection. The consent form, available in both English and Tamil, clearly stated the objectives, procedures, and potential benefits of the study, as well as the right to withdraw at any stage without affecting ongoing medical care. Confidentiality of personal data was strictly maintained, and responses were used solely for research purposes. No financial incentives were offered, and participants were assured that their medical treatment would remain unaffected regardless of participation. The ethical conduct of the study adhered to all institutional and national research guidelines for human subjects.

Data processing and statistical analysis

After data collection, all questionnaires were reviewed for completeness and internal consistency. Data were entered into Microsoft Excel (Redmond, WA, USA) and later imported into SPSS Statistics version 26.0 (IBM Corp., Armonk, NY, USA) for statistical analysis. Descriptive statistics, including frequencies, percentages, means, and standard deviations, were used to summarize socio-demographic data, knowledge levels, and attitudes toward menopause and HRT. Categorical variables such as educational status, marital status, and occupation were analyzed using percentages, while continuous variables like age were presented as mean ± standard deviation.

To determine the relationship between socio-demographic factors and awareness or attitudes toward menopause and HRT, the chi-square (χ²) / Fisher's exact test was applied. A p-value of less than 0.05 was considered statistically significant. Cross-tabulations were used to identify patterns of association between awareness levels and key variables such as age, education, parity, and duration since menopause. Where applicable, logistic regression analysis was planned to identify independent predictors of adequate awareness, though the final inclusion depended on data distribution and variable adequacy.

## Results

Table 1 presents the socio-demographic and menstrual characteristics of the study participants (n = 76). The majority of women were aged 46-50 years (30, 39.5%), followed by those aged above 50 years (26, 34.2%) and 40-45 years (20, 26.3%), representing a typical perimenopausal to postmenopausal age group. With respect to educational attainment, graduates or above (31, 40.8%) constituted the largest group, followed by those who had completed high school (25, 32.9%), primary education (14, 18.4%), and illiterate women (six, 7.9%). Regarding occupational status, the majority were unemployed (48, 63.2%), while employed women (28, 36.8%) formed the remaining proportion. In terms of marital status, married participants (68, 89.5%) predominated, with widowed or divorced women (eight, 10.5%) comprising the rest. Assessment of menstrual status revealed that postmenopausal women (45, 59.2%) - those who had stopped menstruating for more than 12 months - formed the largest group, followed by perimenopausal women with irregular cycles (21, 27.6%) and regularly menstruating women (10, 13.2%).

**Table 1:** Sociodemographic Characteristics of the Study Participants (n=76)

Category	Frequency (n) (n = 76)	Percentage (%)
Age group (in years)		
40–45	20	26.3
46–50	30	39.5
>50	26	34.2
Educational status		
Illiterate	6	7.9
Primary school	14	18.4
High school	25	32.9
Graduate & above	31	40.8
Occupation		
Unemployed	48	63.2
Employed	28	36.8
Marital status		
Married	68	89.5
Widowed/Divorced	8	10.5
Menstrual status		
Still menstruating	10	13.2
Irregular cycles	21	27.6
Stopped >12 months	45	59.2

Table 2 summarizes the participants’ awareness regarding menopause and HRT (n = 76). A large majority of women, 63 (82.9%), reported having heard of the term “menopause,” while 13 (17.1%) had no prior awareness of it. Among those familiar with menopause, 54 participants (71.1%) demonstrated awareness of its common symptoms, such as hot flashes, mood changes, and irregular cycles, whereas 22 women (28.9%) lacked such knowledge. Awareness about HRT was comparatively lower, with only 27 women (35.5%) reporting familiarity, while 49 (64.5%) were unaware of it, indicating a substantial knowledge gap regarding therapeutic options for menopausal management. When asked about their source of information, the most frequently cited medium was media or internet (23, 30.3%), followed by doctors (21, 27.6%), friends or family members (19, 25%), and other sources (13, 17.1%) such as community discussions or health campaigns.

**Table 2:** Awareness of Menopause and HRT Among Participants (n=76) HRT - Hormone replacement therapy

Response	Frequency (n) (n = 76)	Percentage (%)
Heard of “menopause”		
Yes	63	82.9
No	13	17.1
Aware of symptoms of menopause		
Yes	54	71.1
No	22	28.9
Aware of HRT		
Yes	27	35.5
No	49	64.5
Source of information		
Doctor	21	27.6
Friends/Family	19	25
Media/Internet	23	30.3
Others	13	17.1

Table 3 depicts the participants’ knowledge and perceptions regarding health implications and attitudes toward menopause. More than half of the women, 45 (59.2%), correctly recognized that menopause increases the risk of osteoporosis, while 38 (50%) were aware of its association with an elevated risk of cardiovascular disease (CVD), reflecting a moderate understanding of menopause-related health risks. A substantial majority, 60 participants (78.9%), appropriately considered postmenopausal bleeding as an abnormal finding requiring medical evaluation. Interestingly, 30 women (39.5%) perceived menopause as the end of sexual life, while a larger proportion, 46 (60.5%), disagreed, suggesting that most women retained a positive outlook toward sexual well-being in later life. Nearly two-thirds, 49 (64.5%), viewed menopause as a sign of maturity and experience, reflecting cultural acceptance of the transition. Furthermore, 41 participants (53.9%) considered the absence of menstruation a relief, possibly due to the end of menstrual discomfort or inconvenience. Notably, a vast majority, 65 women (85.5%), agreed that menopausal women should consult a physician for guidance and management, underscoring a generally proactive attitude toward health-seeking behavior. The findings also show that 21 women (27%) believed HRT to be safe, while a nearly equal proportion of 20 participants (26%) considered it unsafe. However, a substantial majority of 36 women (47%) were not sure about the safety of HRT, indicating a considerable degree of uncertainty and lack of awareness about the therapy. 

**Table 3:** Knowledge and Attitude Towards Menopause Among the Study Participants CVD - cardiovascular disease

Statement	Yes n (%)	No n (%)
Knows menopause increases risk of osteoporosis	45 (59.2)	31 (40.8)
Knows menopause increases risk of CVD	38 (50.0)	38 (50.0)
Thinks post-menopausal bleeding is abnormal	60 (78.9)	16 (21.1)
Thinks menopause means end of sexual life	30 (39.5)	46 (60.5)
Thinks menopause indicates maturity and experience	49 (64.5)	27 (35.5)
Considers absence of menstruation a relief	41 (53.9)	35 (46.1)
Thinks menopausal women should consult physician	65 (85.5)	11 (14.5)

Table 4 illustrates the association between sociodemographic and awareness variables with knowledge of HRT among the study participants (n = 76). Awareness of HRT was found in 27 women (35.5%), while 49 (64.5%) were not aware of it. There was no significant association between age and HRT awareness (χ² = 0.46, p = 0.79), suggesting that awareness levels were relatively similar across age groups. However, educational status showed a statistically significant association (χ² = 10.72, p = 0.013), indicating that higher education was positively correlated with awareness of HRT - nearly 48.4% of graduates and above were aware compared to only 16.7% of illiterate women. Employment status also demonstrated a significant relationship (χ² = 6.43, p = 0.011), as employed women (60.7%) were more knowledgeable about HRT than those unemployed (20.8%). Additionally, awareness of menopause itself was strongly associated with awareness of HRT (χ² = 4.52, p = 0.034); women who had heard about menopause were significantly more likely to know about HRT than those who had not. These findings highlight that education, employment, and prior knowledge of menopause are key determinants influencing awareness about hormone therapy, whereas age alone does not significantly impact awareness levels. 

**Table 4:** Univariate Analysis of Factors Associated With Awareness of HRT Chi-square/Fisher's exact test *p-value<0.05 is statistically significant HRT - Hormone replacement therapy

Category	Aware of HRT (n=27) n (%)	Not Aware (n=49) n (%)	χ²	p-value
Age group (in years)				
40–45	7 (35.0)	13 (65.0)	0.46	0.79
46–50	11 (36.7)	19 (63.3)		
>50	9 (34.6)	17 (65.4)		
Education				
Illiterate	1 (16.7)	5 (83.3)	10.72	0.013*
Primary school	3 (21.4)	11 (78.6)		
High school	8 (32.0)	17 (68.0)		
Graduate & above	15 (48.4)	16 (51.6)		
Employment				
Unemployed	10 (20.8)	38 (79.2)	6.43	0.011*
Employed	17 (60.7)	11 (39.3)		
Awareness of menopause				
Yes	25 (39.7)	38 (60.3)	4.52	0.034*
No	2 (15.4)	11 (84.6)		

Table 5 presents the results of the multivariate logistic regression analysis examining predictors of awareness about HRT among women aged 40 years and above. After adjusting for potential confounders, educational status, occupation, and awareness of menopause emerged as significant independent predictors of HRT awareness. Women with lower educational attainment (illiterate or schooling level) were significantly less likely to be aware of HRT compared to those with graduate or higher education (Adjusted OR = 0.24, 95% CI: 0.08-0.76, p = 0.015). Similarly, unemployed women had lower odds of being aware of HRT than those who were employed (Adjusted OR = 0.28, 95% CI: 0.10-0.79, p = 0.018), suggesting that employment and exposure to broader social networks may facilitate access to health information. Awareness of menopause also showed a significant association, where women who were not aware of menopause were markedly less likely to know about HRT (Adjusted OR = 0.20, 95% CI: 0.04-0.96, p = 0.043). 

**Table 5:** Multivariate Analysis for Factors Associated With HRT Awareness Among the Study Participants Logistic Regression *p-value<0.05 is statistically significant HRT - Hormone replacement therapy, OR - odds ratio

Variables	Adjusted OR (95% CI)	p-value
Education		
Illiterate / Schooling	0.24 (0.08–0.76)	0.015*
Graduate & above	Ref	
Occupation		
Unemployed	0.28 (0.10–0.79)	0.018*
Employed	Ref	
Awareness of menopause		
Yes	Ref	0.043*
No	0.20 (0.04–0.96)	

**Figure 1:**
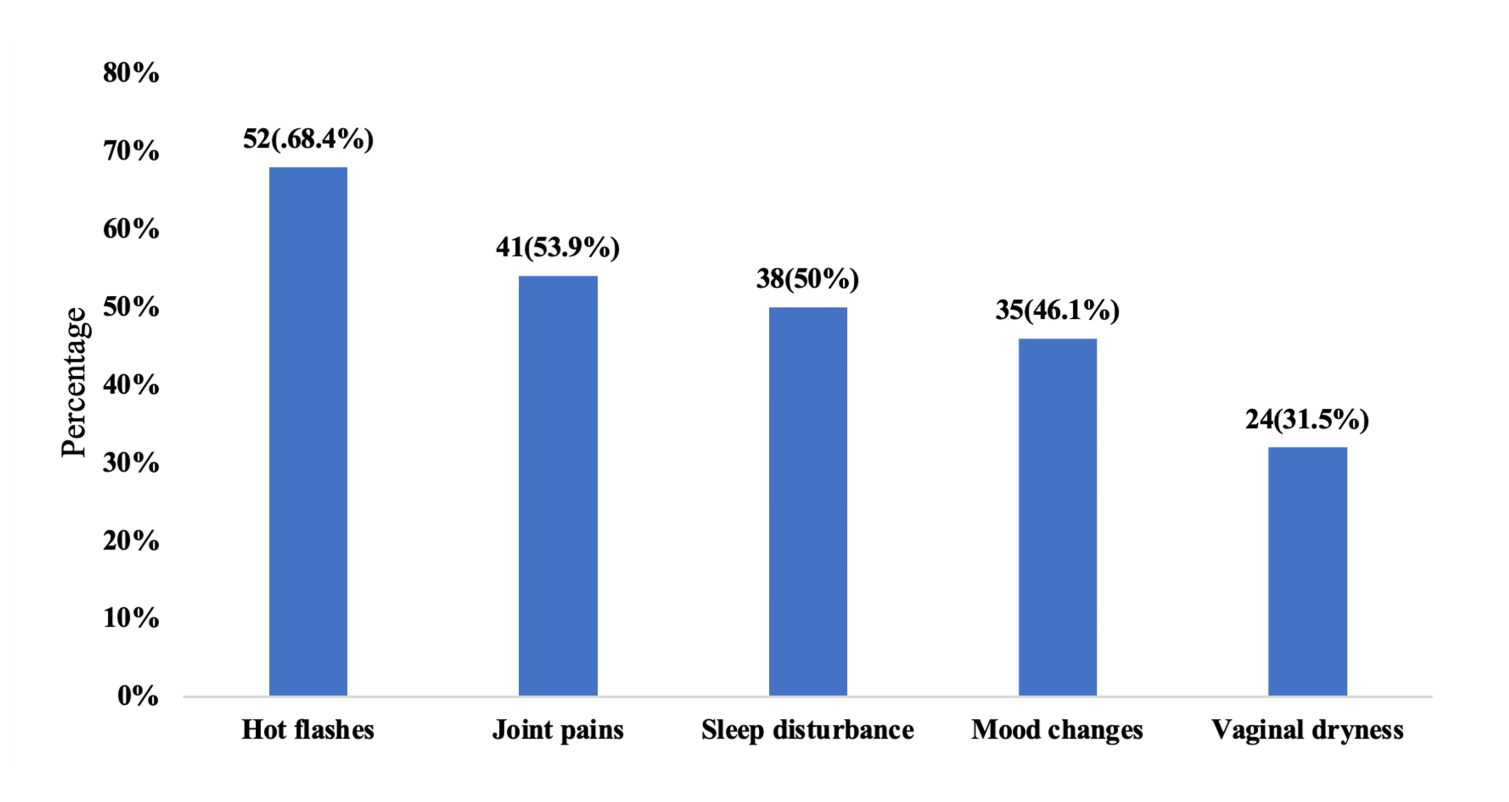
Distribution of Common Menopausal Symptoms Among the Study Participants

Figure 1 illustrates the distribution of common menopausal symptoms among the study participants (n = 76). The most frequently reported symptom was hot flashes, experienced by 52 women (68.4%), followed by joint pains in 41 participants (53.9%). Sleep disturbances were noted in 38 women (50%), while mood changes were reported by 35 participants (46.1%), reflecting the substantial psychosomatic impact of menopause. Vaginal dryness was the least common symptom, affecting 24 women (31.5%). Overall, vasomotor and musculoskeletal complaints were the predominant manifestations, indicating that the majority of women experienced multiple overlapping symptoms during the menopausal transition. 

## Discussion

The present hospital-based cross-sectional study aimed to assess women’s awareness, knowledge, and attitudes regarding menopause and HRT. Overall, the findings highlighted that while awareness of menopause was generally high, knowledge and understanding of HRT remained limited, suggesting a considerable information gap regarding available therapeutic interventions for menopausal symptoms. Most participants were able to identify common menopausal symptoms such as hot flashes and sleep disturbances, indicating that experiential awareness outweighed medical understanding. These observations collectively reflect that although menopause as a concept is well recognized, comprehension of its physiological implications and management options continues to be insufficient.

Comparable findings have been reported in previous research. Rani and Thakur demonstrated that fewer than 40% of women were aware of postmenopausal osteoporosis or its prevention through HRT, revealing persistent misconceptions and a lack of structured education on menopause [15]. Similarly, Sheereen et al. in rural Western Maharashtra found that while a majority of women had heard of menopause, very few knew about available treatment options, underscoring a similar awareness deficit [16]. Mankar et al. also observed that only one-fourth of women sought medical consultation for menopausal complaints, emphasizing the need for proactive health education and physician-led counseling [17]. In South India, Durairaj et al. reported high levels of menopause awareness but low familiarity with HRT, suggesting that literacy and urban exposure may play crucial roles in bridging this gap [18]. Likewise, Akong et al. in Trinidad noted that most women learned about HRT from informal sources such as friends or family rather than healthcare providers, highlighting the missed opportunity for clinicians to act as primary educators [19]. Further, Ang et al., in a multinational study by the Asia-Pacific Menopause Federation, reported low HRT uptake across Asian countries, citing safety concerns and inadequate counseling as major barriers [20].

Education has been consistently identified as a key determinant of menopause and HRT awareness. Aglawe et al. established a positive correlation between educational attainment and knowledge regarding menopause among women in Vidarbha [21]. Similarly, Khalid et al. demonstrated that educational interventions significantly enhanced post-test awareness levels in North Indian women [22]. These findings affirm the pivotal role of education in improving health literacy and enabling informed decision-making regarding menopausal care. Occupational status has also been shown to influence awareness. Hamid et al. in Al-Ain, UAE, observed that employed women had almost double the level of HRT awareness compared to homemakers, suggesting that employment enhances social exposure and access to health information [23]. Such evidence underscores the indirect benefits of women’s empowerment and workforce participation in advancing health-related knowledge.

In terms of attitudes, several studies have documented a mixed perception of menopause. Poomalar and Arounassalame reported that many rural Tamil women viewed menopause as a sign of maturity and freedom from menstrual discomfort, while others associated it with the end of sexual life [24]. Similarly, Borker et al. in Kerala found that while some women perceived menopause as a relief, a comparable proportion experienced emotional distress and psychosocial discomfort [25]. These findings reflect the complex interplay between cultural beliefs, emotional well-being, and health awareness during the menopausal transition. Aaron et al. emphasized that sociocultural taboos and limited communication with healthcare providers hinder women from seeking medical guidance for menopausal concerns, a challenge echoed across multiple regions [26]. Syed Alwi et al. in Malaysia found that although many women had heard of HRT, only a small fraction had discussed it with a physician, reinforcing the need for improved physician-patient dialogue and culturally sensitive counseling approaches [27].

At the international level, similar patterns persist. Jaber et al. in Jordan found that despite a high prevalence of menopausal symptoms, awareness of treatment options remained low [28]. The Asian Menopause Survey conducted by Huang et al. across 11 countries reported that while most women were familiar with menopause, only a minority could accurately describe management strategies [29]. Likewise, John et al. in Gujarat found moderate levels of menopause and HRT awareness accompanied by largely neutral attitudes, a trend consistent across diverse Indian populations [30]. Collectively, these studies underscore the persistent global and regional gaps in menopausal health literacy and the pressing need for structured educational programs and clinician-led interventions to enhance women’s knowledge and attitudes toward menopause and its management. HRT awareness remains low despite high health-seeking behavior, likely because menopause and hormone therapy are rarely discussed during routine consultations, reflecting gaps in provider-initiated counseling. Cultural norms that discourage open conversation about reproductive aging further limit women’s access to accurate information, reducing overall awareness.

Despite the higher awareness levels observed in urban tertiary-care settings, the findings collectively emphasize the need for structured educational interventions at both clinical and community levels. Integrating menopause counseling into routine gynecological visits could bridge the communication gap and enable women to make informed choices regarding symptom management and preventive health strategies. Given that most women relied on informal sources of information, public health campaigns and community education programs could play an instrumental role in dispelling myths surrounding menopause and HRT. The present study had certain limitations. First, the sample size was relatively small (n = 76), which may limit the generalizability of findings beyond the study population. Being a hospital-based and single-centered study, participants likely had higher health literacy than the general population, potentially overestimating awareness levels. The cross-sectional design also restricts causal inference between educational or occupational status and HRT awareness. Furthermore, self-reported data may be subject to recall or social desirability bias. With only 27 HRT-aware participants, logistic regression may be underpowered. The study did not assess longitudinal outcomes such as actual use of HRT or its impact on quality of life. Additionally, the lack of a validated awareness scale may have limited the precision of measurement.

Despite these limitations, the study provides valuable insights into menopausal health awareness among Indian women in a clinical context. The findings highlight the urgent need for integrated health education strategies, including menopause clinics and targeted outreach programs that focus on preventive health, symptom management, and therapeutic literacy. Physicians should actively discuss menopause during midlife consultations, emphasizing the safety and appropriateness of HRT when indicated. Educational outreach through mass media and community-level interventions can address widespread uncertainty and misconceptions. Strengthening awareness initiatives, particularly among less-educated and unemployed women, could promote early consultation, improve quality of life, and empower women to navigate the menopausal transition with confidence and evidence-based understanding.

## Conclusions

In conclusion, the present study highlights that while awareness of menopause among women aged 40 years and above was relatively high, comprehensive knowledge and understanding of HRT remained considerably limited. Education, employment, and prior awareness of menopause were identified as key determinants influencing HRT knowledge, underscoring the importance of socioeconomic and informational empowerment in shaping health literacy. Although the majority of women recognized menopause as a natural transition and were aware of its symptoms, misconceptions regarding its implications and management persist. The findings emphasize the urgent need for structured educational programs, routine counseling during gynecological visits, and greater involvement of healthcare professionals in disseminating accurate information about menopausal health and therapeutic options. Enhancing awareness through culturally sensitive communication and community-based initiatives can bridge existing knowledge gaps, promote positive attitudes, and ultimately improve the quality of life and well-being of midlife women.

## Appendices

Questionnaire: Awareness of Menopause and Hormone Replacement Therapy

Section

Item No.

Question / Variable

Response Options

A. Socio-Demographic Details

1

Age (years)

______

2

Educational Status

Illiterate / Primary / High school / Graduate & above

3

Occupation

Employed / Unemployed

4

Marital Status

Married / Widowed / Divorced

B. Menstrual & Reproductive History

5

Menstrual Status

Still menstruating / Irregular cycles / Stopped >12 months

6

Age at Menopause

______ years

7

Parity

______

C. Awareness of Menopause

8

Heard of menopause

Yes / No

9

Awareness of symptoms of menopause

Yes / No

10

Source of menopause information

Doctor / Friends & Family / Media & Internet / Others

D. Knowledge of Menopausal Health Risks

11

Menopause increases osteoporosis risk

Yes / No

12

Menopause increases cardiovascular disease risk

Yes / No

13

Post-menopausal bleeding is abnormal

Yes / No

E. Attitudes Toward Menopause

14

Menopause means end of sexual life

Agree / Disagree

15

Menopause indicates maturity and experience

Agree / Disagree

16

Absence of menstruation is a relief

Agree / Disagree

17

Menopausal women should consult a physician

Agree / Disagree

F. Awareness of Hormone Replacement Therapy (HRT)

18

Heard of Hormone Replacement Therapy (HRT)

Yes / No

19

Source of HRT information

Doctor / Media & Internet / Friends & Family / Others

20

Know benefits of HRT

Yes / No

21

Know risks of HRT

Yes / No

G. Practices Related to Menopause & HRT

22

Consulted doctor for menopausal symptoms

Yes / No

23

Ever used HRT

Yes / No

24

Reason for HRT use (if applicable)

Symptom relief / Doctor recommendation / Other

25

Reason for not using HRT

Lack of awareness / Fear of side effects / No symptoms / Other
